# Procalcitonin-Guided Strategies for Reducing Antibiotic Use in CAP, HAP, VAP, and COPD: Insights From a Systematic Review and Meta-Analysis

**DOI:** 10.7759/cureus.113973

**Published:** 2026-08-04

**Authors:** Vivek Nangia, Raja Dhar

**Affiliations:** 1 Pulmonology, Institute of Respiratory, Critical Care and Sleep Medicine, Max Hospital, New Delhi, IND; 2 Pulmonology, C K Birla Group of Hospitals, Kolkata, IND

**Keywords:** antibiotic management, cap, clinical outcomes, copd, hap, icu stay, procalcitonin, respiratory infections, vap

## Abstract

Procalcitonin (PCT)-guided therapy may optimize antibiotic use in respiratory infections and chronic lung diseases. This systematic review aimed to assess the role of PCT-guided protocols in reducing antibiotic duration, length of hospital and ICU stay, and clinical outcomes in patients with community-acquired pneumonia (CAP), hospital-acquired pneumonia (HAP), ventilator-associated pneumonia (VAP), and chronic obstructive pulmonary disease (COPD). A secondary literature search was conducted using the databases PubMed and Google Scholar to identify the relevant studies published between January 2003 and December 2023. Eligible studies included peer-reviewed original research (clinical trials, observational, cohort, and case-control studies) evaluating PCT in CAP, VAP, HAP, or COPD, including viral and/or bacterial lower respiratory tract infections (LRTIs) in humans. The excluded studies were animal studies, studies not involving LRTIs, PCT studies lacking extractable data, and non-peer-reviewed editorials, opinions, and conference abstracts. Inconsistency across studies was assessed by heterogeneity analyses and publication bias using funnel plots. Twenty-four studies involving 16,134 patients were included. Across all conditions, PCT-guided therapy reduced antibiotic duration, particularly in CAP, without increasing mortality. Studies included in the CAP group reported that patients in the PCT group benefited from a shorter duration of therapy, with the treatment period decreasing from an average of 13.3±6.5 days in the control group to 11.7±6.2 days in the PCT group. Similarly, the PCT group had significantly reduced ICU stays. For COPD, hospital stay was significantly reduced, though fewer studies demonstrated ICU benefits. PCT-guided protocols may effectively help in better treatment strategies for CAP and COPD management.

## Introduction and background

Respiratory tract infections represent a significant global health challenge, contributing to substantial morbidity and mortality, mainly among populations such as the elderly and individuals with comorbidities. These lower respiratory tract infections encompass hospital-acquired pneumonia (HAP), ventilator-associated pneumonia (VAP), community-acquired pneumonia (CAP), and exacerbations of chronic obstructive pulmonary disease (COPD). HAP and VAP, categorized as nosocomial infections, frequently affect critically ill patients, particularly those requiring mechanical ventilation, leading to increased mortality and heightened healthcare costs due to extended hospitalizations and intensive treatments [1]. Conversely, CAP, a community-onset infection, imposes a dual burden on outpatient and inpatient services globally [2]. COPD is closely linked to lower respiratory tract infections because infective COPD exacerbations, often precipitated by infections, further impair patients' quality of life and drive frequent hospital admissions [3,4]. Prompt and accurate diagnosis, followed by timely initiation of appropriate therapy, remains crucial in these settings, with antibiotics being pivotal in managing bacterial infections. However, the overprescription of antibiotics, particularly in non-bacterial infections, exacerbates the global crisis of antimicrobial resistance [5].

Biomarkers such as procalcitonin (PCT) have gained importance as tools for differentiating bacterial from viral infections, thereby reducing unwarranted antibiotic use. PCT, a peptide precursor of calcitonin, has a low baseline in healthy individuals but markedly increases during systemic bacterial infections, making it an effective diagnostic marker [6]. In patients with lower respiratory tract infection, unlike other inflammatory markers such as C-reactive protein and leukocyte counts, PCT demonstrates higher specificity for infections due to its minimal elevation in viral or non-infectious inflammatory conditions [7-9]. Its rapid decline following effective antibiotic therapy further reinforces its role as a biomarker for diagnosing bacterial infections and monitoring treatment progression [10]. These attributes have prompted investigations into PCT's utility in minimizing unnecessary antibiotic exposure, particularly in respiratory tract infections such as CAP and COPD exacerbations, where viral etiologies are prevalent [11].

The integration of PCT-guided antibiotic therapy for HAP, VAP, CAP, and COPD exacerbations has received increasing clinical interest. While guidelines such as the American Thoracic Society and the Infectious Diseases Society of America recognize the utility of PCT, its role remains supplementary. These emphasize initiating treatment regardless of PCT levels but support PCT-guided antibiotic discontinuation [12]. Numerous studies have explored its diagnostic precision and potential for antibiotic stewardship. For instance, PCT-guided algorithms in CAP patients have reduced antibiotic usage without adversely affecting clinical outcomes [13]. Similarly, in COPD exacerbations, shorter antibiotic courses with fewer adverse effects have been observed with PCT guidance [14]. However, its widespread clinical adoption is hindered by inconsistent findings across studies, attributed to variations in PCT cutoff thresholds, time of level measurement, and population-specific applications [15]. Furthermore, while PCT is highly effective in identifying bacterial infections, its capacity to predict infection severity and its applicability in non-bacterial COPD exacerbations remain debatable.

This systematic review aims to address these gaps by evaluating evidence on the use of PCT-guided treatment for HAP, VAP, CAP, and COPD exacerbations. By collating available data, this review seeks to elucidate the clinical benefits of PCT-guided treatment, particularly its potential to optimize antibiotic use, improve patient outcomes, and mitigate antimicrobial resistance.

## Review

Methodology

This systematic review aims to evaluate the role of PCT-guided therapy in reducing the duration of antibiotic treatment and length of stay in the intensive care unit (ICU) and hospital for patients with lower respiratory tract infection, namely CAP, HAP, VAP, and COPD. The review will assess whether PCT-guided protocols lead to more efficient antibiotic management compared to standard of care, and if these strategies affect the overall clinical outcomes. The secondary endpoint was the comparison of clinical outcomes, namely mortality, between PCT-guided therapy and standard care.

Search Strategy

The PICO (Patient/Population, Intervention, Comparator/Control, and Outcome(s)) approach was used to synthesize the research question and guide the literature search followed by analysis (Table 1) [16].

**Table 1:** PICO criteria [14] PICO: Patient/Population, Intervention, Comparator/Control, and Outcome(s); CAP: community-acquired pneumonia, HAP: hospital-acquired pneumonia, VAP: ventilator-associated pneumonia, and chronic obstructive pulmonary disease (COPD).

	Inclusion criteria
Population	Patients diagnosed with any one of the respiratory tract infections namely: HAP/VAP/CAP/COPD.
Intervention/exposure	Procalcitonin-guided antibiotic treatment
Comparator	Antibiotic treatment
Outcomes	Primary outcomes: Duration of antibiotic treatment Secondary outcomes: Length of ICU/Hospital stay Exploratory Outcome: Clinical Outcomes and Mortality

We conducted a comprehensive literature search in PubMed and Google Scholar to identify studies on the role of procalcitonin in lower respiratory tract infections (LRTIs). The search included articles published between January 2003 and December 2023, used a combination of Medical Subject Headings (MeSH) and free-text terms such as "Procalcitonin," " hospital-acquired pneumonia” (HAP), “ventilator-associated pneumonia” (VAP), “community-acquired pneumonia” (CAP), and “chronic obstructive pulmonary disease” (COPD). Boolean operators (AND, OR) optimized the search. Additionally, we reviewed the reference lists of relevant articles to capture any missed studies.

Inclusion and Exclusion Criteria

Studies were selected based on pre-specified inclusion and exclusion criteria. The inclusion criteria were: (1) original research articles reporting on the role of PCT in CAP/VAP/HAP/COPD, including viral and bacterial infections; (2) studies conducted in humans; (3) randomized controlled trials, observational studies, cohort studies, and case-control studies; (4) articles published in English.

The exclusion criteria included: (1) animal model studies or studies not involving lower respiratory tract infections (LRTIs); (2) studies focusing on PCT but lacking study-related data points; (3) non-peer-reviewed literature such as editorials, opinion pieces, and conference abstracts.

Data Extraction

Two reviewers independently extracted data, including study design, population details, type of LRTI (HAP/VAP, CAP, COPD), PCT levels, antibiotic-free days, hospital stay, mortality, and relevant outcomes. Data extraction was performed using a standardized Excel (Microsoft, Redmond, WA) sheet. Primary and secondary outcomes were predefined, and all compatible results across reported measures and time points were collected. Additional data included the study design, participant diagnoses, study population size (N), intervention or exposure details, and follow-up duration. Missing or unclear information was handled using predefined assumptions or excluded from quantitative analysis when necessary.

Data Analysis

Where data allowed, meta-analysis was considered to pool effect sizes from studies reporting similar outcomes using weighted mean differences or relative risks with 95% confidence intervals (RevMan software, The Cochrane Collaboration, London, UK). We used a web-based Cochrane Risk of Bias tool (robvis) for the studies included in this meta-analysis [17]. Individual study results and pooled estimates were tabulated and displayed using forest plots. Publication bias was checked using funnel plots for datasets with over 10 studies.

Ethical Considerations

Since this review involved publicly available literature, no ethical approval was required. The study adhered to the Preferred Reporting Items for Systematic Reviews and Meta-Analyses (PRISMA) guidelines to ensure transparency (Table 3 in the Appendices).

Results

**Figure 1:**
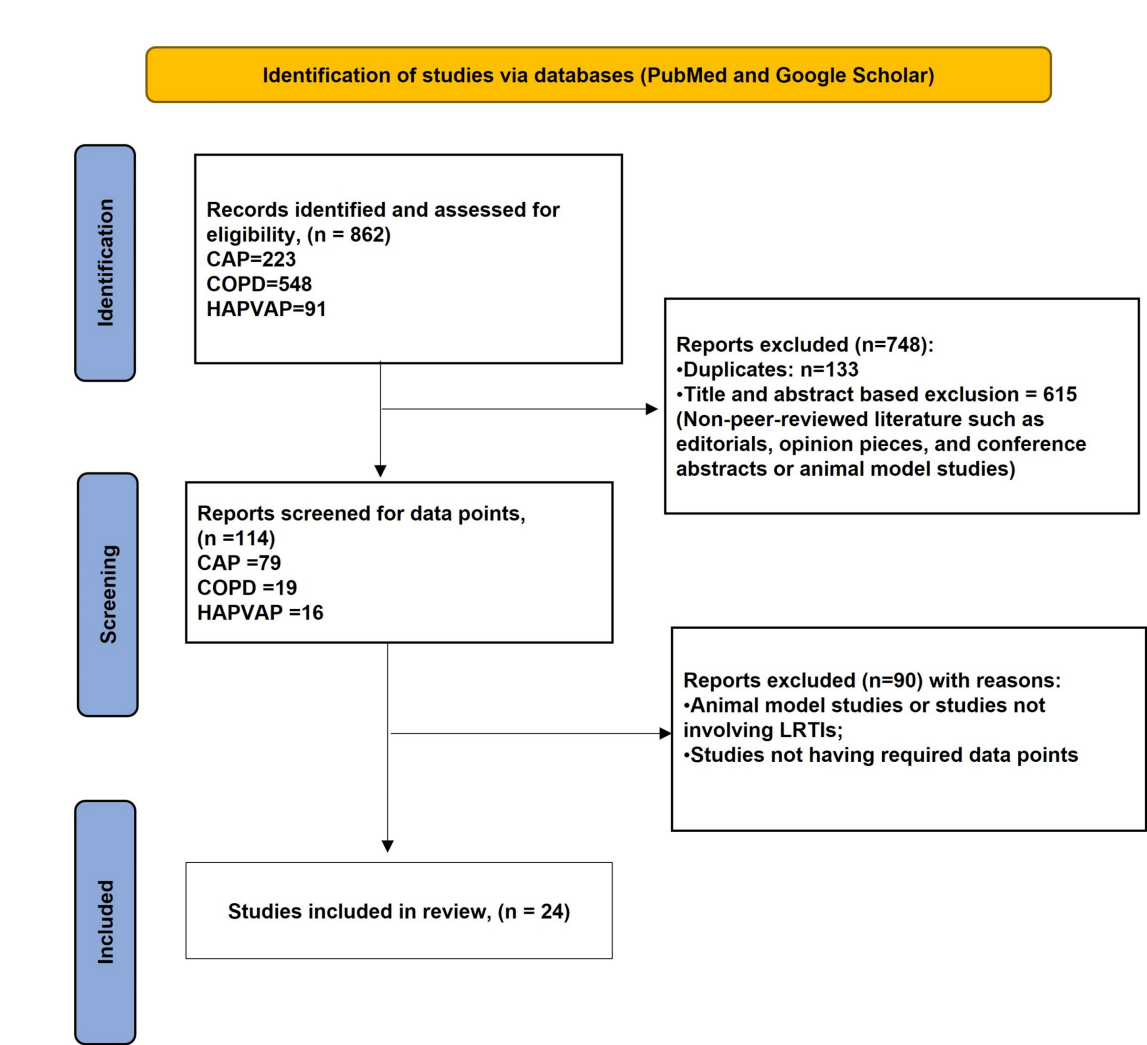
Study flow diagram CAP: Community-acquired pneumonia; COPD: chronic obstructive pulmonary disease; HAP: hospital-acquired pneumonia; LTRI: lower respiratory tract infection; VAP: ventilator-associated pneumonia.

A total of 114 articles were initially shortlisted. Out of these, 24 studies fulfilled the selection criteria (Figure 1) and are included in this meta-analysis. Table 2 outlines the characteristics of the included studies along with the bifurcation according to the disease conditions, namely, CAP, COPD, and HAP/VAP. 

**Table 2:** The characteristics of the included studies

S. No.	Study	Disease	Population, N	Study Type	Location
1	Scheutz et al. 2018 [7]	CAP	6708	Randomized	Not Mentioned
2	Scheutz et al. 2009 [18]	CAP/COPD	1359	Randomized	Switzerland
3	Christ-Crain et al. 2004 [19]	CAP/COPD	243	Randomized	Switzerland
4	Christ-Crain et al. 2006 [11]	CAP	302	Randomized	Switzerland
5	Briel et al. 2008 [20]	CAP	458	Randomized	Switzerland
6	Kristoffersen et al. 2009 [21]	CAP/COPD	210	Randomized	Denmark
7	Stolz et al. 2009 [22]	CAP/HAP/VAP/COPD	101	Randomized	USA
8	Bukhardt et al. 2010 [23]	CAP	550	Observational	Not Mentioned
9	Annane et al. 2013 [24]	CAP	62	Randomized	France
10	Bouadma et al. 2010 [25]	CAP/HAP/VAP	621	Randomized	France
11	de Jong et al. 2016 [26]	CAP	1546	Randomized	Netherland
12	Deliberato et al. 2013 [27]	CAP	81	Randomized	Brazil
13	Hochreiter et al. 2009 [28]	CAP	110	Randomized	Heide
14	Layios et al. 2012 [29]	CAP	509	Randomized	Belgium
15	Khajavi et al. 2015 [30]	CAP	60	Randomized	Iran
16	Nobre et al. 2008 [31]	CAP	79	Randomized	Switzerland
17	Schroeder et al. 2009 [32]	CAP	27	Randomized	Heide
18	Shehabi et al. 2014 [33]	CAP	394	Randomized	Australia
19	Corti et al. 2016 [34]	COPD	120	Randomized	Bielenberg
20	Liu and Zhang 2015 [35]	COPD	108	Randomized	Not mentioned
21	Nangia and Gandhi 2012 [36]	COPD	100	Randomized	Not mentioned
22	Verduri et al. 2015 [37]	COPD	183	Randomized	Italy
23	Wang et al. 2016 [38]	COPD	194	Randomized	Beijing
24	Daubin et al. 2018 [39]	COPD	302	Randomized	France

Antibiotic Duration

**Figure 2:**
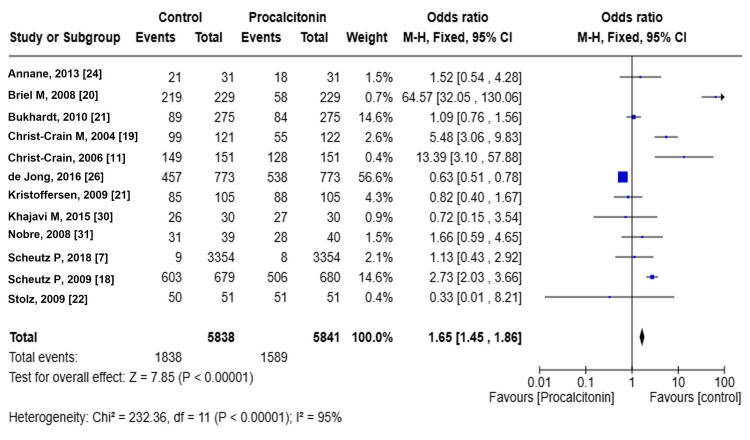
Forest plot for duration of antibiotic therapy for studies including patients with community-acquired pneumonia

**Figure 3:**
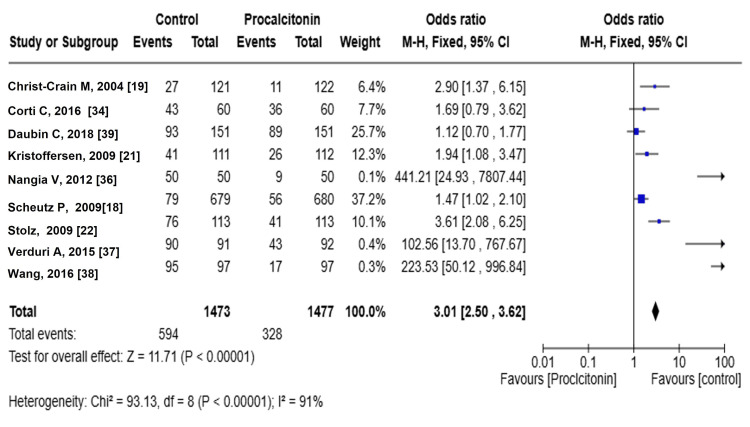
Forest plot for duration of antibiotic therapy for studies including patients with chronic obstructive pulmonary disease

This analysis evaluated 12 studies enrolling 5838 patients with CAP in the control group and 5841 patients with CAP in the PCT-guided treatment group. Additionally, nine studies enrolling patients with COPD (n=1473, control group, and n=1477, PCT group) were also analyzed. The analysis of these studies showed that PCT-guided therapy significantly reduced the duration of antibiotic treatment compared to standard care in patients with CAP and COPD. In studies evaluating patients with CAP, patients in the PCT group experienced reduced therapy duration from 13.3±6.5 days (mean±SD) in the control group to 11.7±6.2 days in the PCT group. The use of PCT may help in earlier discontinuation of antibiotics by indicating when bacterial infections are unlikely, thereby shortening unnecessary antibiotic exposure and reducing the risk of antimicrobial resistance (Figures 2, 3).

Length of Hospital Stay

**Figure 4:**
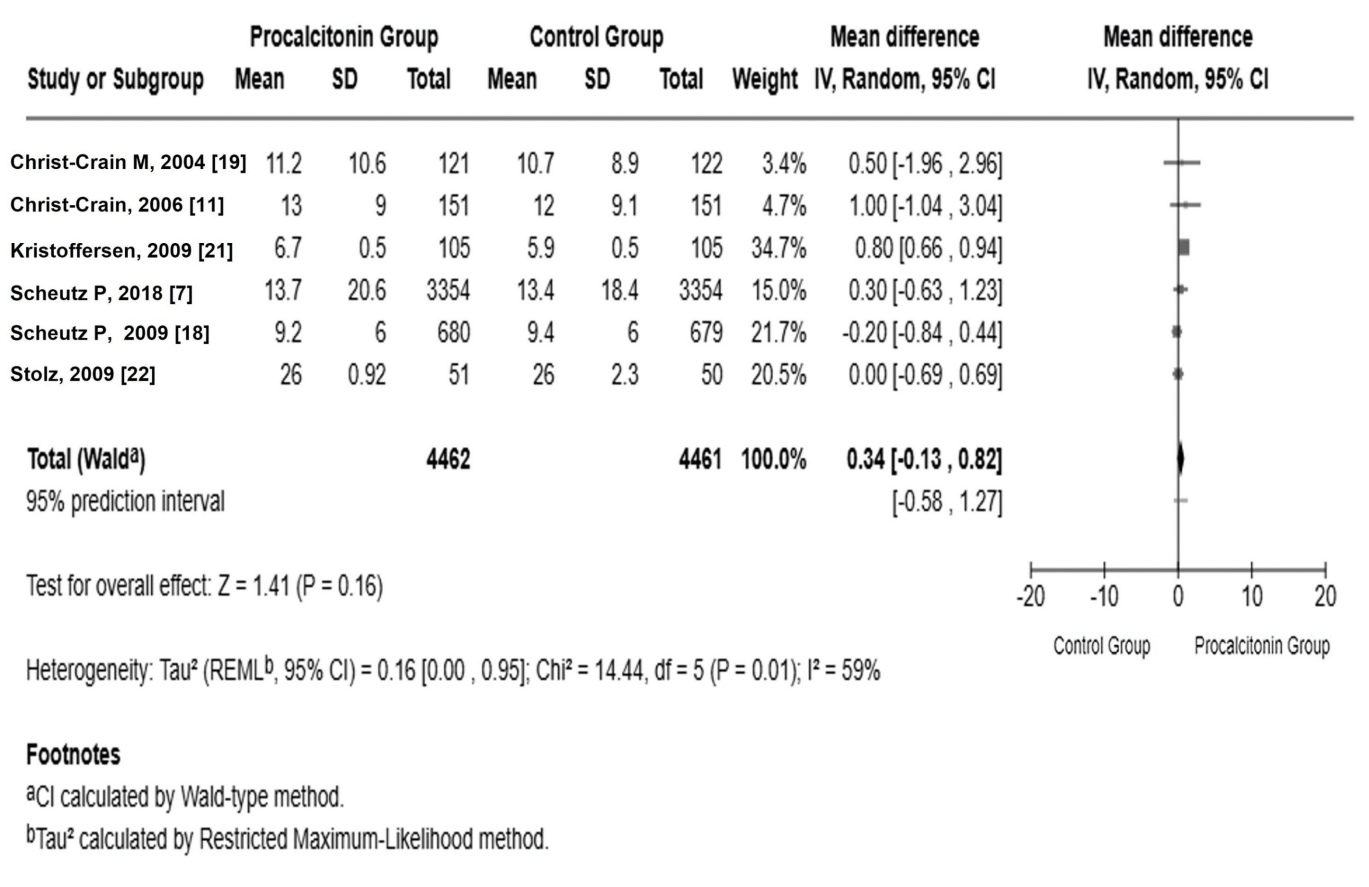
Forest plot for length of hospital stay for studies including patients with community-acquired pneumonia

Six studies focused on CAP were analysed for the length of hospital stay between PCT-guided therapy and standard care groups. The overall effect size (Z=1.14, p=0.16) showed no significant difference between the PCT and control groups. The heterogeneity was low (I²=59%), indicating variation across the studies. The forest plot depicts the diamond overlapping the line of no effect, suggesting that PCT-guided therapy did not significantly reduce the length of hospital stay compared with standard care (Figure 4).

**Figure 5:**
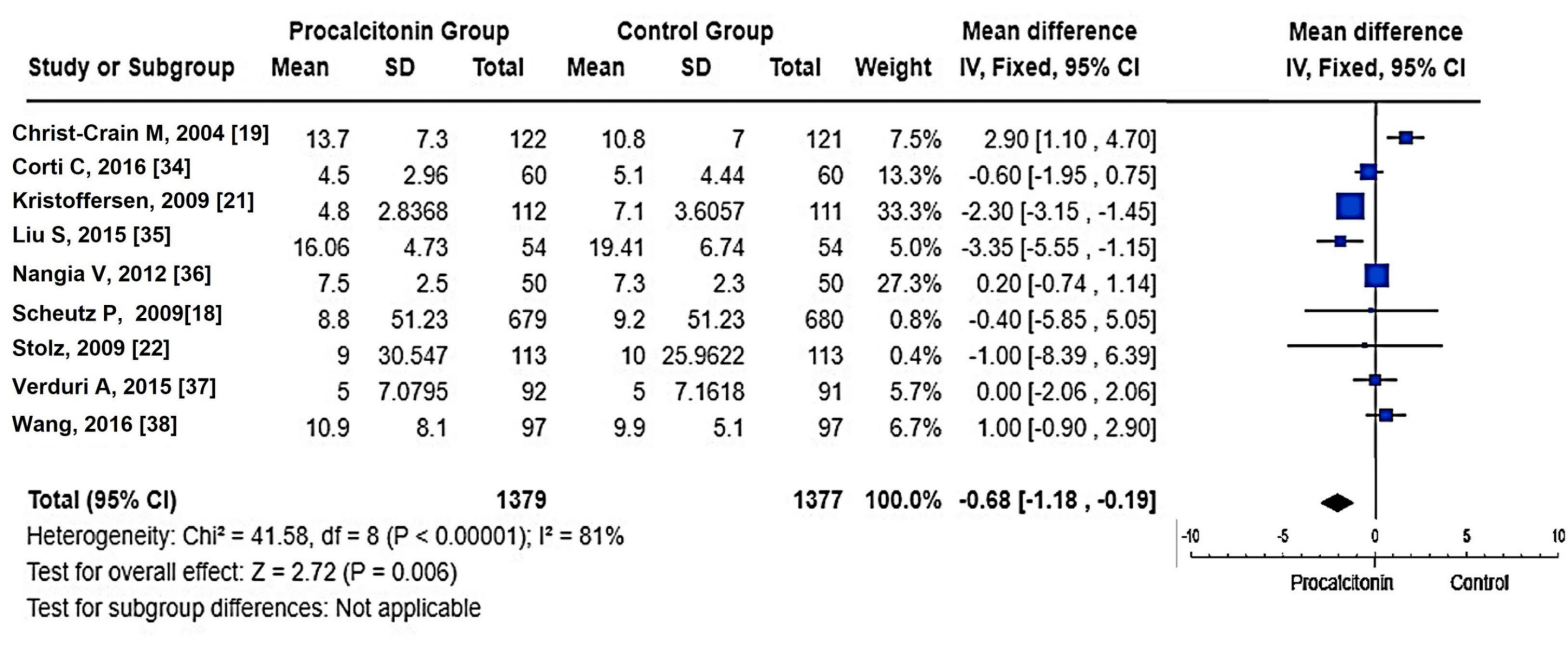
Forest plot for hospital stay for studies including patients with chronic obstructive pulmonary disease

Nine studies involving COPD patients demonstrated a significant reduction in hospital stay for those managed with PCT guidance. The overall effect size was statistically significant (Z=2.72, p=0.006). However, there was substantial heterogeneity (I²=81%), indicating variability among the studies. The forest plot showed a clear distinction between the two arms, with the line of no effect not crossing the diamond, confirming the effectiveness of PCT-guided therapy in reducing hospital stay for COPD patients (Figure 5).

Length of ICU Stay

**Figure 6:**
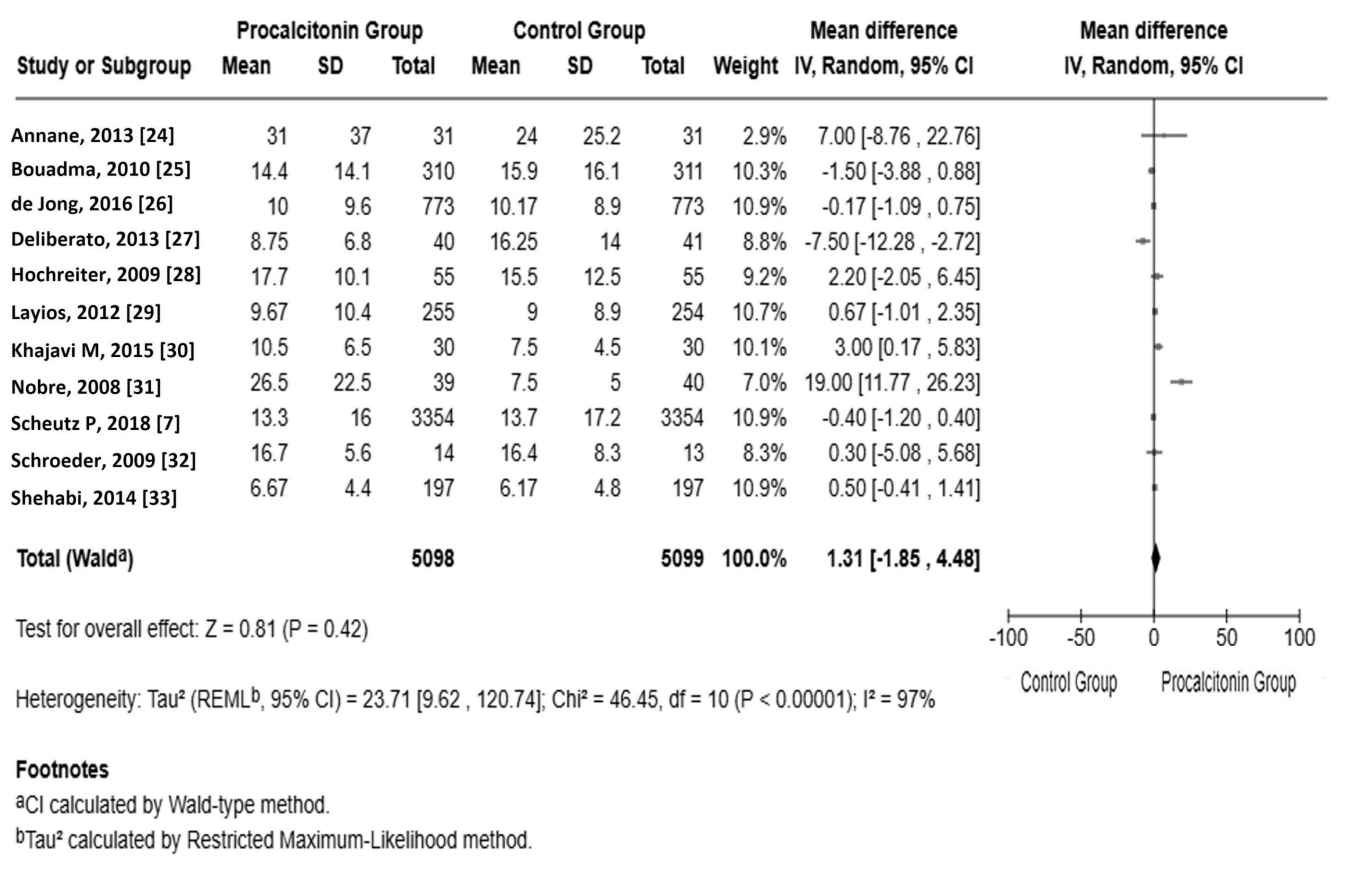
Forest plot for length of ICU stay for studies including patients with community-acquired pneumonia

Eleven studies assessing ICU length of stay in CAP patients reported a reduction in ICU duration in the PCT-guided group (z=0.81, p=0.42) and significant heterogeneity (I²=97%). The forest plot indicated that the line of no effect did not cross the diamond, reflecting a reduction in ICU stay for the PCT group (Figure 6).

In COPD patients, only one of the studies included in this analysis indicated a significant reduction in ICU length of stay (days), mean±SD, for survivors managed with PCT guidance being 16±15 vs. 25±17 for non-survivors. Two studies focused on HAP/VAP patients did not show a significant reduction in either hospital or ICU stay with PCT-guided therapy. The odds ratio for hospital stay was 0.99 (95% CI: 0.62-1.56, p=0.95), and for ICU stay, the odds ratio was 0.87 (95% CI: 0.42-1.81, p=0.71). Both results showed no significant difference between the PCT and control groups.

Clinical Outcomes and Mortality

**Figure 7:**
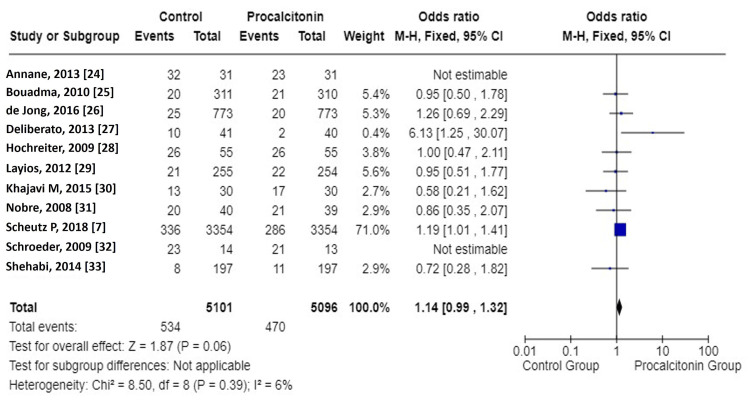
Forest plots for 30-day mortality for studies including patients with community acquired pneumonia

The overall mortality rates between PCT-guided therapy and standard care showed no significant differences across CAP, HAP/VAP, or COPD patients. As shown in Figure 7 for CAP patients, the odds ratio for 30-day mortality was 1.14 (p=0.06), indicating no statistically significant difference in survival.

**Figure 8:**
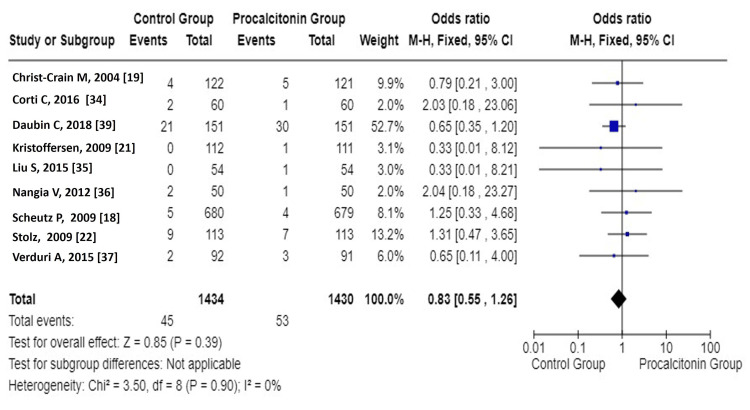
Overall mortality in patients with chronic obstructive pulmonary disease

Similarly, in the COPD cohort (Figure 8), no significant difference in mortality was observed (p=0.39). For HAP/VAP patients, no significant differences were found in 28- or 60-day mortality (p=0.92 and p=0.37, respectively). These findings highlight the trend of reduced antibiotic use and duration of ICU stays with PCT guidance without compromising patient survival outcomes.

Risk of Bias Assessment

**Figure 9:**
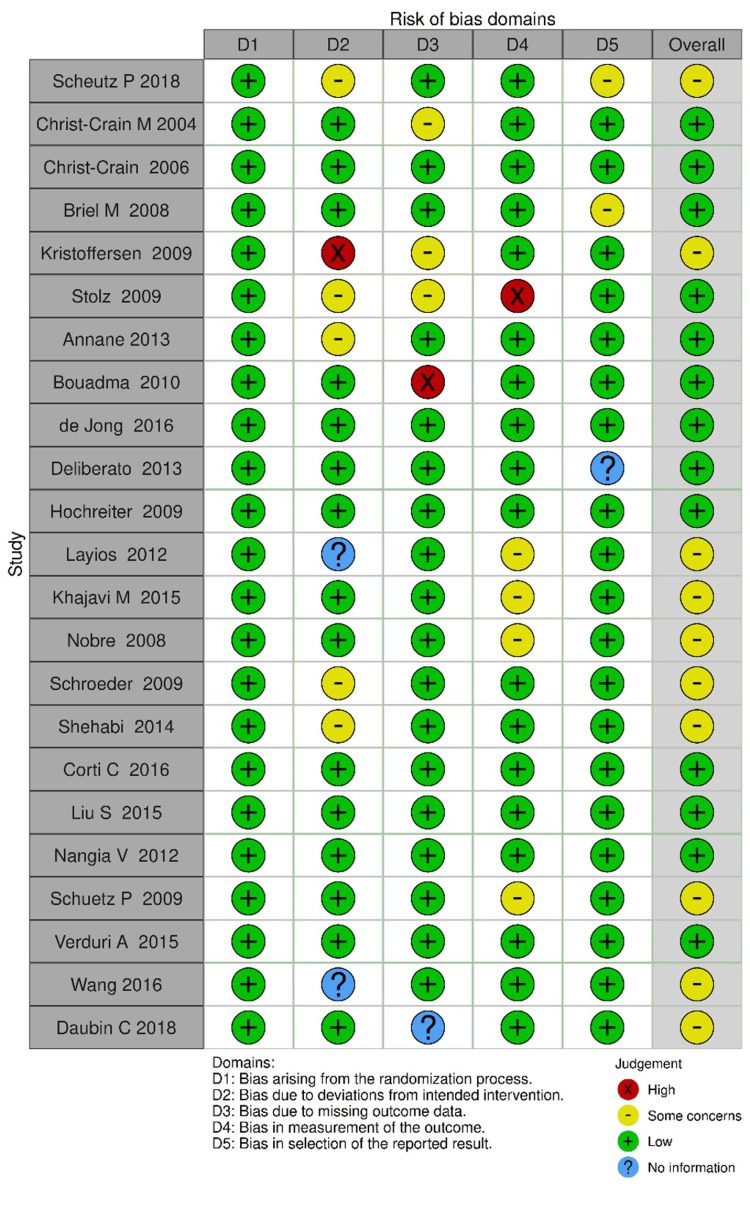
Risk of bias assesment for randomised studies included in this meta-analysis This is a traffic light plot generated by Robvis, a web-based tool for visualizing risk-of-bias assessments [7,9,15-19,21-36].

Risk of bias was assessed using the Cochrane Risk of Bias tool for randomized trials (RoB 2). Twenty-three out of the 24 included studies were randomized controlled studies (RCTs) and were analysed using this tool. The robvis online tool was also used for visualizing the results of the risk of bias assessments. The overall publication risk bias is low or has very few concerns, as shown in the traffic light display in Figure 9.

**Figure 10:**
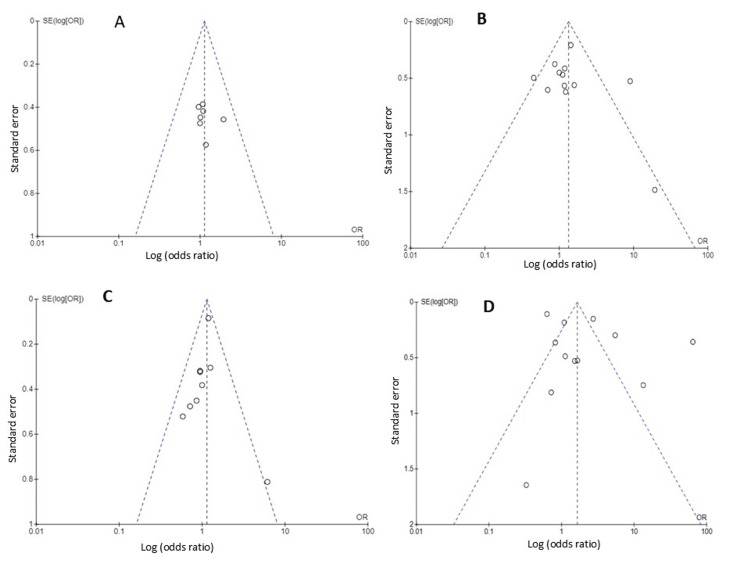
Publication bias for studies under community-acquired pneumonia (CAP) A. Length of stay (days) in hospital; B. Length of stay (days) in ICU; C. 30-Day mortality; D. Antibiotic therapy duration.

**Figure 11:**
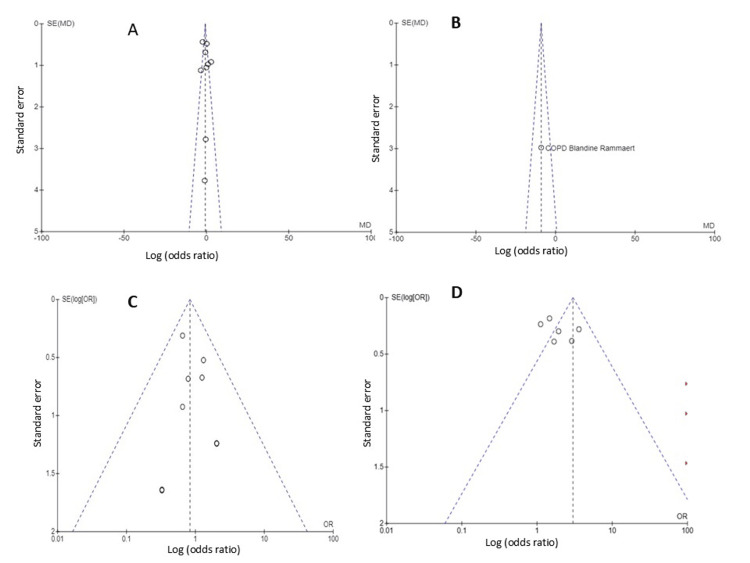
Publication bias for studies under chronic obstructive pulmonary disease (COPD) publication bias for studies under COPD A. Length of stay (days) in hospital; B. Length of stay (days) in ICU; C. Overall mortality; D. Antibiotic therapy duration.

**Figure 12:**
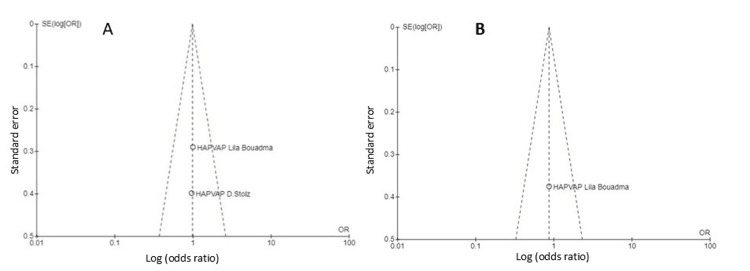
Publication bias for studies under HAP/VAP Analysis A. Length of stay (days) in hospital for control and procalcitonin group; B. Length of stay (days) in ICU for both the groups.

The CAP studies (n>10) indicated no major publication bias, as evidenced by the symmetrical distribution of studies in the funnel plots (Figure 10 in the Appendices). Studies with smaller sample sizes were evenly distributed at the bottom of the plot, while larger studies were clustered at the top, confirming that the results were not significantly influenced by bias. Similarly, the funnel plot for COPD studies also revealed no significant publication bias (Figure 11 in the Appendices). However, for HAP/VAP studies, given the limited number of trials (n=2), publication bias analysis was not applicable (Figure 12 in the Appendices).

Discussion

The findings of this systematic review and meta-analysis demonstrate that PCT-guided therapy reduces antibiotic duration and ICU stays in patients with CAP and COPD, without compromising clinical outcomes. This aligns with previous studies supporting PCT as an effective biomarker for antibiotic stewardship, particularly in bacterial respiratory infections, where overuse of antibiotics can lead to resistance and adverse effects [40]. A recent multi-centre randomized controlled trial [25] confirmed that PCT-guided therapy reduced antibiotic use by approximately 1.67 days, and extended antibiotic-free days by 2.26 days (95% CI 1.40-3.12), highlighting the biomarker’s value in optimizing antibiotic regimens [25].

In CAP patients, the reduction in the length of hospital and ICU stay suggests that PCT, in addition to standard clinical assessments, enables early and accurate decision-making regarding the discontinuation of antibiotics. However, length of hospital and ICU stay did not show a significant reduction, likely reflecting variability in institutional discharge policies or recovery outside the ICU [24]. These findings are consistent with several large-scale trials that have reported the benefits of PCT in limiting unnecessary antibiotic use without negatively impacting clinical outcomes [40,41].

Conversely, in patients with HAP and VAP, PCT did not significantly reduce antibiotic duration or ICU stay. This is possibly due to the complexities involved in treating these critically ill patients, including the prevalence of multidrug-resistant organisms, which often require extended antibiotic courses beyond what PCT can guide [25]. Critically ill patients, especially those on ventilators, may have multifactorial conditions that limit the utility of a single biomarker such as PCT, necessitating comprehensive clinical judgement [40].

The significant reduction in antibiotic-related side effects in both CAP and COPD patients (p=0.0001) further underscores the utility of PCT-guided therapy. This reduction in side effects is crucial for improving patient safety and minimizing the risks associated with prolonged antibiotic use, such as drug toxicity [41]. Furthermore, PCT’s role in mitigating antimicrobial resistance by avoiding unnecessary antibiotic use aligns with global efforts to curb the rise of resistant pathogens [42].

While these results are promising, several limitations must be acknowledged. The heterogeneity observed in hospital stay durations, particularly for COPD patients, indicates variability across studies that may stem from differences in healthcare settings, treatment protocols, or patient characteristics. Additionally, many of the included studies did not adequately control for confounding factors such as disease severity, comorbidities, and variations in antibiotic regimens, which may influence the outcomes independently of PCT levels. The inability to analyse publication bias in HAP/VAP due to a limited number of studies further limits the generalizability of the findings for this subgroup.

## Conclusions

This review systematically evaluates the role of PCT in guiding antibiotic therapy across multiple respiratory conditions, including CAP, COPD, and HAP/VAP. By synthesizing the evidence, this study demonstrates PCT’s efficacy in reducing antibiotic exposure and ICU stay, particularly in CAP and COPD, without compromising clinical outcomes. These findings support the integration of PCT as an adjunct biomarker into clinical protocols for respiratory infections, although more research is needed to explore its role in HAP/VAP further and to standardize its application across diverse healthcare settings.

## Appendices

**Table 3:** PRISMA 2020 Checklist PRISMA: Preferred Reporting Items for Systematic Reviews and Meta-Analyses.

Section and Topic	Item #	Checklist item	Location where item is reported
TITLE			
Title	1	Identify the report as a systematic review.	Line 1-2
ABSTRACT			
Abstract	2	See the PRISMA 2020 for Abstracts checklist.	Line 4-24
INTRODUCTION			
Rationale	3	Describe the rationale for the review in the context of existing knowledge.	Line 39-66
Objectives	4	Provide an explicit statement of the objective(s) or question(s) the review addresses.	Line 67-70
METHODS			
Eligibility criteria	5	Specify the inclusion and exclusion criteria for the review and how studies were grouped for the syntheses.	Line 80-95
Information sources	6	Specify all databases, registers, websites, organisations, reference lists and other sources searched or consulted to identify studies. Specify the date when each source was last searched or consulted.	Line 81-87
Search strategy	7	Present the full search strategies for all databases, registers and websites, including any filters and limits used.	Line 82-95
Selection process	8	Specify the methods used to decide whether a study met the inclusion criteria of the review, including how many reviewers screened each record and each report retrieved, whether they worked independently, and if applicable, details of automation tools used in the process.	Line 96-103
Data collection process	9	Specify the methods used to collect data from reports, including how many reviewers collected data from each report, whether they worked independently, any processes for obtaining or confirming data from study investigators, and if applicable, details of automation tools used in the process.	Line 96-103
Data items	10a	List and define all outcomes for which data were sought. Specify whether all results that were compatible with each outcome domain in each study were sought (e.g. for all measures, time points, analyses), and if not, the methods used to decide which results to collect.	Line 81, Table 1, Line 99-103
	10b	List and define all other variables for which data were sought (e.g. participant and intervention characteristics, funding sources). Describe any assumptions made about any missing or unclear information.	Line 104-109
Study risk of bias assessment	11	Specify the methods used to assess risk of bias in the included studies, including details of the tool(s) used, how many reviewers assessed each study and whether they worked independently, and if applicable, details of automation tools used in the process.	Line 105-109
Effect measures	12	Specify for each outcome the effect measure(s) (e.g. risk ratio, mean difference) used in the synthesis or presentation of results.	Line 106-110
Synthesis methods	13a	Describe the processes used to decide which studies were eligible for each synthesis (e.g. tabulating the study intervention characteristics and comparing against the planned groups for each synthesis (item #5)).	Line 106-110
	13b	Describe any methods required to prepare the data for presentation or synthesis, such as handling of missing summary statistics, or data conversions.	Line 106-110
	13c	Describe any methods used to tabulate or visually display results of individual studies and syntheses.	Line 106-110
	13d	Describe any methods used to synthesize results and provide a rationale for the choice(s). If meta-analysis was performed, describe the model(s), method(s) to identify the presence and extent of statistical heterogeneity, and software package(s) used.	Line 106-110
	13e	Describe any methods used to explore possible causes of heterogeneity among study results (e.g. subgroup analysis, meta-regression).	Line 106-110
	13f	Describe any sensitivity analyses conducted to assess robustness of the synthesized results.	Line 106-110
Reporting bias assessment	14	Describe any methods used to assess risk of bias due to missing results in a synthesis (arising from reporting biases).	Line 106-110
Certainty assessment	15	Describe any methods used to assess certainty (or confidence) in the body of evidence for an outcome.	Line 106-110
RESULTS			
Study selection	16a	Describe the results of the search and selection process, from the number of records identified in the search to the number of studies included in the review, ideally using a flow diagram.	116-119
	16b	Cite studies that might appear to meet the inclusion criteria, but which were excluded, and explain why they were excluded.	Figure 1
Study characteristics	17	Cite each included study and present its characteristics.	Table 2
Risk of bias in studies	18	Present assessments of risk of bias for each included study.	Supplementary material, Figure 5
Results of individual studies	19	For all outcomes, present, for each study: (a) summary statistics for each group (where appropriate) and (b) an effect estimate and its precision (e.g. confidence/credible interval), ideally using structured tables or plots.	Figures 1-5
Results of syntheses	20a	For each synthesis, briefly summarise the characteristics and risk of bias among contributing studies.	Supplementary material, Figure 5, line 162-169
	20b	Present results of all statistical syntheses conducted. If meta-analysis was done, present for each the summary estimate and its precision (e.g. confidence/credible interval) and measures of statistical heterogeneity. If comparing groups, describe the direction of the effect.	Figures 1-5 and Line 116-161
	20c	Present results of all investigations of possible causes of heterogeneity among study results.	Figures 1-5
	20d	Present results of all sensitivity analyses conducted to assess the robustness of the synthesized results.	Figures 1-5
Reporting biases	21	Present assessments of risk of bias due to missing results (arising from reporting biases) for each synthesis assessed.	Supplementary material, Figure 5
Certainty of evidence	22	Present assessments of certainty (or confidence) in the body of evidence for each outcome assessed.	Supplementary material, Figure 5
DISCUSSION			
Discussion	23a	Provide a general interpretation of the results in the context of other evidence.	Line 171-195
	23b	Discuss any limitations of the evidence included in the review.	Line 196-201
	23c	Discuss any limitations of the review processes used.	Line 196-202
	23d	Discuss implications of the results for practice, policy, and future research.	Line 204-209
OTHER INFORMATION			
Registration and protocol	24a	Provide registration information for the review, including register name and registration number, or state that the review was not registered.	Not registered
	24b	Indicate where the review protocol can be accessed, or state that a protocol was not prepared.	NA
	24c	Describe and explain any amendments to information provided at registration or in the protocol.	NA
Support	25	Describe sources of financial or non-financial support for the review, and the role of the funders or sponsors in the review.	Line 210
Competing interests	26	Declare any competing interests of review authors.	Line 211
Availability of data, code and other materials	27	Report which of the following are publicly available and where they can be found: template data collection forms; data extracted from included studies; data used for all analyses; analytic code; any other materials used in the review.	Line 212-213
